# 

**Published:** 1877-10

**Authors:** 


### Table of Contents.

### ORIGINAL COMMUNICATIONS.

Inhalation of Iodine in Asthma. *By J. G. Westmoreland, M. D., Atlanta, Ga* ........ 385

A Case of Stricture of the Urethra, complicated with Hypertrophy of the Prostate, Stone and Violent Cystitis. *By Shirley Bragg, M. D., Atlanta, Ga* ........ 388

Nephritic Colic. *By Ferdinand King, M. D., Atlanta, Ga* ........ 391

Botany in its Relations with Medicine, No. 1. *By W. T. Grant, M. D., Atlanta, Ga.* ........ 393

Is Bromide Potass. and Sulph. Morphia an Antidote for Poisoning by Strychnine? *By J. T. Moore, M. D., Cairo, Ga* ........ 396

Introductory Lecture to the Course of 1877-78 in Atlanta Medical College. *By J. G. Westmoreland, M. D., Atlanta, Ga* ........ 397

### REPORTS OF SOCIETIES.

Atlanta Academy of Medicine ........ 403

Toledo Medical Association ........ 410

Cincinnati Academy of Medicine ........ 412

### SELECTIONS.

The Operative Treatment of Genu-Valgum ........ 414

Retention of Fractural Maxillæ ........ 416

An Interesting case of Obstetrics, with General Remarks ........ 417

Charlatantry in Destructive Diseases ........ 419

Glycerite of Kephaline ........ 422

Recent Progress in Syphilology ........ 426

### EDITORIAL.

American Pharmaceutal Association ........ 432

Medical Colleges ........ 436

Editorial Correspondence ........ 436, 439, 440

European Correspondence ........ 441

### BIBLIOGRAPHICAL.

Outlines of Modern Chemistry, Organic ........ 446

Hospitals: Their History, Organization and Construction ........ 447

### EXCERPTA.

Nerve Stretching in Neuralgia ........ 447

Bromide of Lithium ........ 448

